# The Feature of Distribution and Clonality of TCR **γ**/**δ** Subfamilies T Cells in Patients with B-Cell Non-Hodgkin Lymphoma

**DOI:** 10.1155/2014/241246

**Published:** 2014-05-21

**Authors:** Liang Wang, Meng Xu, Chunyan Wang, Lihua Zhu, Junyan Hu, Shaohua Chen, Xiuli Wu, Bo Li, Yangqiu Li

**Affiliations:** ^1^Department of Oncology, First Affiliated Hospital, Jinan University, Guangzhou 510632, China; ^2^Centre of Oncology and Hematology, First Affiliated Hospital of Guangzhou Medical College, Guangzhou 510230, China; ^3^Department of Rheumatism and Immunology, First Affiliated Hospital, Jinan University, Guangzhou 510632, China; ^4^Institute of Hematology, Jinan University, Guangzhou 510632, China; ^5^Key Laboratory for Regenerative Medicine of Ministry of Education, Jinan University, Guangzhou 510632, China

## Abstract

Restricted T-cell receptor (TCR) V*α*/V*β* repertoire expression and clonal expansion of *αβ* T cells especially for putative tumor-associated antigens were observed in patients with hematological malignancies. To further characterize the *γδ* T-cell immune status in B-cell non-Hodgkin lymphoma (B-NHL), we investigated the distribution and clonality of TCR V*γ*/V*δ* repertoire in peripheral blood (PB), bone marrow (BM), and lymph node (LN) from patients with B-NHL. Four newly diagnosed B-NHL cases, including three with diffuse large B-cell lymphoma (DLBCL) and one with small lymphocytic lymphoma (SLL), were enrolled. The restrictive expression of TCR V*γ*/V*δ* subfamilies with different distribution patterns could be detected in PB, BM, or LN from all of four patients, and partial subfamily T cells showed clonal proliferation. At least one clonally expanded V*δ* subfamily member was found in PB from each patient. However, the expression pattern and clonality of TCR V*γ*/V*δ* changed in different immune organs and showed individual feature in different patients. The clonally expanded V*δ*5, V*δ*6, and V*δ*8 were detected only in PB but neither in BM nor LN while clonally expanded V*δ*2 and V*δ*3 could be detected in both PB and BM/LN. In conclusion, the results provide a preliminary profile of distribution and clonality of TCR *γ*/*δ* subfamilies T cells in PB, BM, and LN from B-NHL; similar clonally expanded V*δ* subfamily T cells in PB and BM may be related to the same B-cell lymphoma-associated antigens, while the different reactive clonally expanded V*γ*/V*δ* T cells may be due to local immune response.

## 1. Introduction

B-cell non-Hodgkin lymphoma (B-NHL) is a heterogeneous group of malignant lymphoproliferative disorders originating in B lymphocytes, which comprises approximately 80–85% of newly diagnosed cases with NHL. Although current therapeutic strategies, including standard chemotherapy, unlabeled or radiolabeled monoclonal antibodies, high-dose chemotherapy following autologous peripheral blood stem cell transplantation, or allogeneic hematopoietic stem cell transplantation, have significantly improved the outcome of this disease, the majority of patients relapse or become resistant to prior therapies. Therefore, novel strategies, such as cellular immunotherapy, are increasingly investigated [1].

Poor cellular immune function may relate to carcinogenic processes and to worse prognosis in tumor patients. Moreover, the progression of tumor might further induce the cellular immune suppression. In recent years, molecular analysis of the T-cell receptor (TCR) utilization feature, based on the principle of TCR *α*, *β*, *γ*, and *δ* gene rearrangement and deletion rearrangement, has proven to be an effective technique for studying the distribution of T cell repertoire, the diversity of TCR subfamilies [2, 3], the antigen specific expansion of T-cell clones, and the recent thymic output function [4, 5]. This in turn can help to characterize the feature of host T-cell immune status and the identification of T-cell populations of interest in cancer, as well as the peripheral immune repertoire reconstitution after hematopoietic stem cell transplantation (HSCT).

T cells possessing a *γδ* TCR are a small subset of human T cells (1–10% of all peripheral blood T cells). These cells share effector functions with *αβ* T cells as well as with natural killer (NK) cells, particularly the capacity to interact with dendritic cells (DCs) [6, 7]. Mice deficient in *γδ* T cells show a significantly increased incidence of tumors and provide clear evidence for an immune surveillance function of these unconventional lymphocytes [8]. Human V*γ*9V*δ*2 T cells can kill a broad spectrum of tumor cells with or without reduced MHC class I molecules expression in an MHC-unrestricted manner [9–14]. Moreover, *γδ* T cells can migrate as infiltrating lymphocytes into solid tumors [9] and can recognize and eliminate cultured malignant cells (primary cells or cell lines) from myeloma [10, 11], non-Hodgkin lymphoma [12], prostate cancer [13], renal cell carcinoma [14], colon carcinoma [15], and squamous cell carcinoma [16]. Obviously, *γδ* T cells play an important role in immunosurveillance and anticancer response and become more and more attractive for cell therapy strategies against cancer. However, little is known about *γδ* T-cell immune status in B-NHL patients. Bartkowiak et al. have reported that the highly restricted TCR V*γ* subfamily usage that is predominant for TCR V*γ*II (V*γ*9) was characterized in chronic lymphocytic leukemia (CLL) [17].

T cells recognize specific ligands by specific TCRs, which are glycoprotein heterodimers comprising either *α*/*β* or *γ*/*δ* chains. Rearrangement of the individual variable (V), diversity (D), joining (J), and constant (C) regions leads to the creation of the hypervariable complementarity determining region 3 (CDR3) of the functional TCR, which plays a pivotal role in the recognition of antigenic epitopes [18, 19]. *γδ* T cells rearrange and express clonally diverse antigen receptors in a manner similar to *αβ* T lymphocytes; however, the V, D, and J element repertoire in the TCR *γ* and TCR *δ* loci is limited in number. The TCR *γ* gene contains at least 14 functional variable (TCR V*γ*) segments belonging to four subgroups (i.e., TCR V*γ*I to IV), and the TCR *δ* gene contains at least eight functional TCR V*δ* segments that are subdivided into eight V*δ* subfamilies (i.e., V*δ*1–V*δ*8). Our previous study showed that restricted TCR V*α* and V*β* repertoire expression and clonal expansion of *αβ* T cells were observed in peripheral blood from patients with diffuse large B-cell lymphoma (DLBCL) [20]. To further characterize the *γδ* T-cell immune status in B-NHL, we investigated the distribution and clonality of TCR V*γ* and V*δ* repertoire in peripheral blood (PB), bone marrow (BM), and lymph node (LN) from patients with B-NHL.

## 2. Materials and Methods

### 2.1. Samples

Four male patients with B-NHL diagnosed according to the World Health Organization (WHO) criteria were enrolled in the present study (designated as C1–C4). The clinical characteristics of these patients are described in Table 1. After the patient's consent, PB samples were obtained from all of the four patients, BM samples were obtained from 3 (C1, C2, and C3) of the four patients, and lymphoma-infiltrated LN samples were obtained from diagnosed biopsy in 3 patients (C1, C2, and C4), respectively. All procedures were conducted according to the guidelines of the medical ethics committees of the Health Bureau of Guangdong Province, China.

### 2.2. Mononuclear Cells Isolation, RNA Isolation, and cDNA Synthesis

Mononuclear cells of PB or BM samples (PBMCs or BMMCs) were isolated by Ficoll-Hypaque gradient centrifugation. RNA was extracted from PBMCs, BMMCs, or LN homogenate using a RNA extraction buffer according to the manufacturer's protocol (Trizol, Invitrogen, USA). The RNA quality was analyzed in 0.8% agarose gel stained with ethidium bromide. Two *μ*g RNA was reversely transcribed into the first single-strand cDNA with random hexamer primers, using reverse transcriptase, Superscript II Kit (Gibco, USA). The cDNA quality was confirmed by RT-PCR for *β*2 microglobulin gene amplification.

### 2.3. RT-PCR for the TCR V*γ* and TCR V*δ* Subfamily Amplification

Three sense TCR V*γ* primers and a single TCR C*γ* reverse primer or eight TCR V*δ* sense primers and a single TCR C*δ* primer were used in unlabeled PCR for amplification of the TCR V*γ* and V*δ* subfamilies, respectively. Subsequently, a run-off PCR was performed with fluorescent primers labeled at 5′ end with the FAM fluorophore (C*γ*-FAM or C*δ*-FAM) (TIB MOLBIOL GmbH, Berlin, Germany). The sequences of primers are listed in Table 2. The PCR was performed as previously described [21]. Aliquots of the cDNA (1 *μ*L) were amplified in 20 *μ*L mixtures with one of the three V*γ* primers and a C*γ* primer or one of the eight V*δ* primers and a C*δ* primer. The final reaction mixture contained 0.5 *μ*M of the sense and antisense primers, 0.1 mM dNTPs, 1.5 mM MgCl_2_, 1 × PCR buffer, and 1.25 U Taq polymerase (Promega, USA). The amplification was performed in a DNA thermal cycler (BioMetra, Germany) with 3 min denaturation at 94°C and 40 PCR cycles. Each cycle consisted of 94°C for 1 min, 60°C for 1 min, and 72°C for 1 min, respectively, and a final 7 min elongation at 72°C. The PCR products were stored at 4°C and ready for genescan analysis.

### 2.4. Genescan Analysis for TCR V*γ* and TCR V*δ* Subfamily Clonality

Aliquots of the unlabeled PCR products (2 *μ*L) were subjected to a cycle of run-off reaction with fluorophore-labeled C*γ*-FAM or C*δ*-FAM primer, respectively. The labeled runoff PCR products (2 *μ*L) were heat denatured at 94°C for 4 min with 9.5 *μ*L formamide (Hi-Di Formamide, ABI, USA) and 0.5 *μ*L of size standards (Genscan-500-LIZ, Perkin Elmer, ABI). The samples were then loaded into a 3100 POP-4 gel (Performance Optimized Polymer-4, ABI, USA) and resolved by electrophoresis in a 3100 DNA sequencer (ABI, Perkin Elmer) for size and fluorescence intensity determination using Genescan software [21]. Since the positions of the V*γ*/V*δ* and C*γ*/C*δ* primers are fixed, the length distribution observed in the PCR V*γ*-C*γ*/V*δ*-C*δ* products depends only on the size of the rearrangement of V-J (in TCR*γ*) or V-D, D-J (in TCR*δ*) gene segment and the randomly inserted nucleotides (VN(DN)J). After electrophoresis on an automated sequencer and subsequent computer analysis, the products of different size could be separated and expressed as different peaks.

## 3. Results

### 3.1. The Distribution and Clonally Expanded TCR V*γ* and V*δ* T Cells in Peripheral Blood from Patients with B-NHL

**Figure 1:**
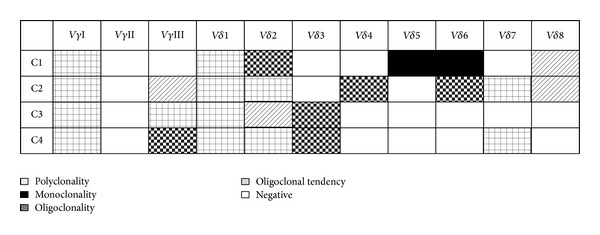
The distribution and clonality features of TCR V*γ* and V*δ* subfamilies in PBMCs from four patients with B-NHL (C1–C4).

The expression of TCR V*γ* and V*δ* subfamilies was detected by RT-PCR, and no PCR products were scored as negative for the corresponding TCR subfamily by agarose gel electrophoresis. Only one or two of the three TCR V*γ* subfamilies and four to six of the eight TCR V*δ* subfamily members could be detected in PB samples. V*γ*I, V*δ*1, and V*δ*2 were expressed in all samples, whereas V*γ*II expression was absent in all of the four samples. In addition, V*γ*III (3/4), V*δ*3 (2/4), V*δ*4 (1/4), V*δ*5 (1/4), V*δ*6 (2/4), V*δ*7 (2/4), and V*δ*8 (2/4) could be detected in partial cases (Figure 1).

**Figure 2:**
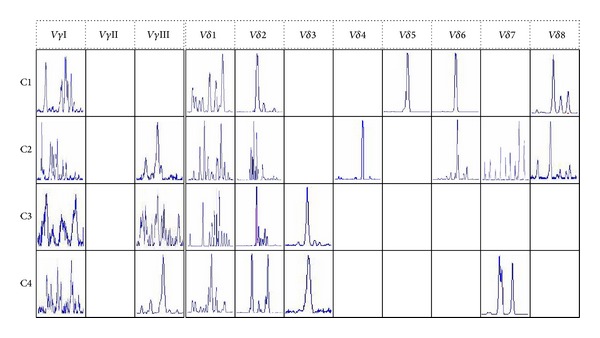
The results of Genescan of TCR V*γ* and V*δ* subfamilies in PBMCs from four patients with B-NHL (C1–C4).

The clonality of TCR V*γ* and V*δ* subfamily T cells was identified by Genescan analysis, which was used to identify the CDR3 size and assess the spectratype pattern visually. Polyclonality of TCR V*γ* and V*δ* subfamily T cells displays a Gaussian or nearly Gaussian-like distribution consisting of multiple peaks (usually 6–8), and any deviation from the Gaussian profile (skewed repertoire) would indicate clonally expanded pattern. Oligoclonality and biclonality shows a skewed spectratype profile with a single dominant peak and double peaks, respectively. Oligoclonal trending is between polyclonal and oligoclonal. The PCR product analysis produces only one peak, which means that CDR3 lengths are identical, named as the monoclonal pattern [21, 22]. For the TCR V*γ* and V*δ* subfamily clonality analysis, only the clonally expanded V*γ*III subfamily was detected in samples from C2 and C4 cases, whereas clonally expanded V*δ* T cells could be identified in almost all subfamilies (except for V*δ*1) in different patients. Additional, at least one clonally expanded TCR V*δ* subfamily member were found in PB in every patient. The distribution of the clonally expanded *γδ* T cells in PB is shown in Figures 1 and 2.

### 3.2. Different Expression Patterns of TCR V*γ* and V*δ* in Peripheral Blood, Bone Marrow, or Lymph Node

**Figure 3:**
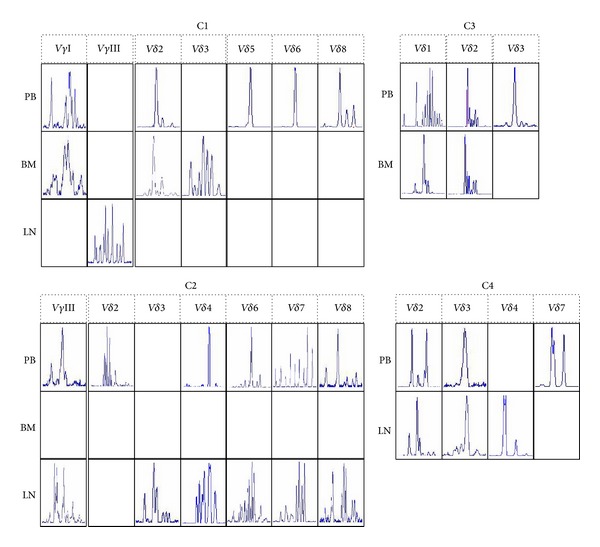
Different expression patterns of TCR V*γ* and V*δ* in peripheral blood (PB), bone marrow (BM), or lymph node (LN) from B-NHL cases (C1–C4).

The expressions of V*γ*II/V*δ*4/V*δ*5/V*δ*6/V*δ*7/V*δ*8 and V*γ*II/V*δ*5 were absent in BM and LN samples, respectively. Clonal expansion of TCR V*δ* repertoire could be found in some TCR V*δ* subfamilies in PB, BM, or LN, which displayed different patterns in different patients (Figure 3). In patient C1, the clonally expanded V*δ*2, V*δ*5, V*δ*6, and V*δ*8 subfamily T cells could be found in PB, whereas only the clonally expanded V*δ*2 could be detected in BM, and there has been no clonal expansion of TCR V*γ*/V*δ* subfamilies in LN. In patient C2, the clonality of V*δ*4, V*δ*6, and V*δ*8 changed from oligoclonality or oligoclonal trend in PB to polyclonality in LN, whereas the expression of those was absent in BM. In patient C3, the clonally expanded V*δ*2/V*δ*3 and V*δ*1/V*δ*2 T cells were found in PB and BM, respectively. In patient C4 with SLL, the clonally expanded V*δ*3 and V*δ*2/V*δ*3 T cells were found in PB and LN, respectively. Interestingly, the oligoclonally expanded V*δ*3 T cells could be found in PB from patients C3 and C4 and in LN from patient C4.

## 4. Discussion

Analysis of alterations in the TCR repertoire is an effective investigational approach that may help to understand involved immunological abnormalities and provide guidance for clinical applications using this information [23, 24]. Recent data indicated that T-cell immunodeficiency is a common feature in different hematological malignancies, including the absence of TCR V*α* and V*β* subfamilies, decreased diversity of TCR repertoires, reduced thymic recent output function (naïve T cells), and lower frequencies of TCR subfamily naïve T cells [25]. Apart from *αβ* T cells, *γδ* T cells also play important roles in immunosurveillance and anticancer response. Different TCR V*γ* and V*δ* subfamily expression patterns have been reported in patients with leukemia, myelodysplastic syndrome (MDS), and immune thrombocytopenic purpura (ITP) [3, 17, 26, 27]. However, little is known about the distribution and clonality of the TCR V*γ* and V*δ* subfamilies in B-cell lymphoma. In the present study, we analyzed the distribution and clonal expansion of TCR V*γ* and V*δ* T cells in four B-NHL patients and compared the different expression patterns of TCR V*γ* and V*δ* in peripheral blood, bone marrow, or lymph node in individual cases. In contrast to healthy individuals and patients with MDS or ITP previously reported [26, 27], the absence of TCR V*γ*II subfamily was found in all blood, marrow, and lymph node samples from B-NHL patients, which imply a widespread restricted TCR V*γ* repertoire expression pattern may be a feature in patients with B-NHL. Moreover, the distribution of V*γ* and V*δ* subfamilies was not identical in samples between peripheral blood, bone marrow, or lymph node, and this may be due to the distribution or expansion of *γδ* T cells in different immune organs, however, further investigation is needed to summarize this feature in a large cohort samples and follow up the change of *γδ* T-cell repertoire on the outcome of the patients.

Like the change of clonality of TCR subfamily in leukemia [17, 27], clonally expanded TCR V*δ* subfamilies could be found in peripheral blood from all of four B-NHL patients, which is thought to be related to the tumor associated antigens [17, 20, 27]. In this study, we analyzed the distribution of clonally expanded V*δ* T cells not only in peripheral blood, but also in bone marrow or lymph node, we were interested to find out the identical expanded V*δ* T cell clones, similar clonal expanded V*δ*2 subfamily T cells were detected in both peripheral blood and bone marrow samples in two cases with DLBCL, and similar clonality of V*δ*3 subfamily T cells was identified in peripheral blood and lymph node in one case with SLL; these preliminary data suggested that these V*δ* T-cell clones might respond to the same B-cell lymphoma-associated antigens. However, different reactive clonally expanded V*δ* T cells between peripheral blood, bone marrow, and lymph node may be due to local immune response. Further investigation is needed to determine whether these clonally expanded T cells are related to antilymphoma cells.

In addition, in contrast to the clonally expanded V*δ*2, V*δ*3, and V*δ*4 T cells in lymph node from the SLL patient (C4), none clonal expansion of TCR V*γ* or V*δ* T cells was detected in lymphoma cell-infiltrated lymph node samples donated by the two cases with DLBCL (C1 and C2), suggesting that the deficiency of clonal expansion of *γδ* T cells in lymphoma cell-infiltrated lymph node may be another feature in DLBCL. However, whether it is related to tumor microenvironments of lymph node remains an open question and needs to further explore.

## 5. Conclusion 

In this study, we characterized the distribution and clonality of V*γ* and V*δ* repertoire in peripheral blood, bone marrow, and lymph node from B-NHL patients, we found obviously different features of restrictive usage and clonal proliferation of TCR V*γ* and V*δ* subfamilies in individual patients as well as in different immune organs; even if we found some identical clonally expanded V*δ* subfamily T cells in peripheral blood and bone marrow, similar clonal expanded V*δ* subfamily T cells in peripheral blood and bone marrow may be related to the same B-cell lymphoma-associated antigens, while the different reactive clonally expanded V*γ*/V*δ* T cells may be due to local immune response. However, whether it is related to different antigen stimulation and tumor microenvironments remains an open question and needs to further explore.

## Figures and Tables

**Table 1:** Clinical characteristics of the included patients with B-NHL.

Patient number	Gender	Age (yrs)	Disease subtype	Ann Arbor staging
C1	Male	57	DLBCL	IVA
C2	Male	60	DLBCL	IVB
C3	Male	56	DLBCL	IVA
C4	Male	76	SLL	IIIB

DLBCL: diffuse large B-cell lymphoma; SLL: small lymphocytic lymphoma; yrs: years.

**Table 2:** List of primer sequences used in this study.

Primer	Sequence
V*γ*I	5′-TACCTACACCAGGAGGGGAAG-3′
V*γ*II	5′-GGCACTGTCAGAAAGGAATC-3′
V*γ*III	5′-TCGACGCAGCATGGGTAAGAC-3′
C*γ*	5′-GTTGCTCTTCTTTTCTTGCC-3′
C*γ*-FAM	5′-FAM-CATCTGCATCAAGTTGTTTATC-3′
V*δ*1	5′-GTGGTCGCTATTCTGTCAACT-3′
V*δ*2	5′-GCTCCATGAAAGGAGAAGCGA-3′
V*δ*3	5′-CACTGTATATTCAAATCCAGA-3′
V*δ*4	5′-TGACACCAGTGATCCAAGTTA-3′
V*δ*5	5′-TCTGCACATTGTGCCCTCCCA-3′
V*δ*6	5′-TATCATGGATTCCCAGCC-3′
V*δ*7	5′-GAACATCACAGCCACCCAGACCG-3′
V*δ*8	5′-ACTTCCAGAAAGCAGCCAAA-3′
C*δ*	5′-AACAGCATTCGTAGCCCAAGCAC-3′
C*δ*-FAM	5′-FAM-GTTTATGGCAGCTCTTTGAAGGT-3′
